# Fecal Microbiota Composition as a Metagenomic Biomarker of Dietary Intake

**DOI:** 10.3390/ijms24054918

**Published:** 2023-03-03

**Authors:** Nathalia Caroline de Oliveira Melo, Amanda Cuevas-Sierra, Edwin Fernández-Cruz, Victor de la O, José Alfredo Martínez

**Affiliations:** 1 IMDEA-Food Institute (Madrid Institute for Advances Studies), Campus of International Excellence (CEI), 28040 Madrid, Spain; 2 Centro de Investigación Biomédica en Red Fisiopatología de la Obesidad y la Nutrición (CIBEROBN), Instituto de Salud Carlos III, 28049 Madrid, Spain

**Keywords:** biomarker, dietary patterns, fidelity measures, food intake, gut microbiota, precision nutrition

## Abstract

Gut microbiota encompasses the set of microorganisms that colonize the gastrointestinal tract with mutual relationships that are key for host homeostasis. Increasing evidence supports cross intercommunication between the intestinal microbiome and the eubiosis–dysbiosis binomial, indicating a networking role of gut bacteria as potential metabolic health surrogate markers. The abundance and diversity of the fecal microbial community are already recognized to be associated with several disorders, such as obesity, cardiometabolic events, gastrointestinal alterations, and mental diseases, which suggests that intestinal microbes may be a valuable tool as causal or as consequence biomarkers. In this context, the fecal microbiota could also be used as an adequate and informative proxy of the nutritional composition of the food intake and about the adherence to dietary patterns, such as the Mediterranean or Western diets, by displaying specific fecal microbiome signatures. The aim of this review was to discuss the potential use of gut microbial composition as a putative biomarker of food intake and to screen the sensitivity value of fecal microbiota in the evaluation of dietary interventions as a reliable and precise alternative to subjective questionnaires.

## 1. Introduction

Investigations about the influence of nutrition on human health are crucial to understand the pivotal involvement of food intake consumption on the prevention, development, and management of chronic diseases, such as obesity or type 2 diabetes [1,2,3]. In this context, dietary intervention often needs to measure nutrient intake as well as to monitor the adherence of patients to nutritional prescriptions, whose assessment or control may provide reliability and precision in metabolic management. In nutritional practice, dietary evaluation is usually performed via traditional methods: diet recall, diet diaries, or food frequency questionnaires; these supply information about nutrient consumption [4]. Available methods about food intake measurements are frequently implemented in dietetic applications, whose advantages include the relatively easy data collection and the possibility of rapid verification of the adherence to nutritional interventions at low cost. However, these methods present limitations related to the ability to accurately assess food intake. Complementarily, in the last few years, there has been an increasing interest in the use of blood and urinary determinations as food intake biomarkers [5], while fecal microbiota is envisaged to have a role based on metagenomic approaches [6].

Indeed, modern dietary biomarkers involve measurable and quantifiable metabolic determinations, which can be evaluated in different biological samples that also potentially identify physiological processes related to food intake of a nutrient or dietary pattern, reflecting a more precise dietetic assessment [7]. Additionally, multiple factors need to be necessarily considered to establish an ideal biomarker of food intake, as concerns specificity, sensibility, and plausibility. Furthermore, a characteristic response over time and dose after food intake is expected, as well as being reproducible with a specific food group. Chemically, the biomarker should be stable in the selected matrix, during sample analysis and along storage, and the analytical technique to identify must be inexpensive, as far as possible. Moreover, factors related with the biomarker and analytical methods, such a robustness and reliability, should be addressed during method validation, followed by analytical performance parameters, such as limits of detection and quantification, precision, and accuracy [8]. Although defining all factors related to an ideal biomarker is difficult, it is highly recommended to fulfill as many viable conditions as possible before selecting a potential candidate as a food intake biomarker.

Noteworthily, in the era of ‘omics’ technology, biomarkers that suitably estimate intake foods or dietary patterns are scarce. The lack of effective and accurate biomarkers makes it difficult to perform studies requiring this information and make it necessary to rely on participant subjective recall, which often produces biases. Diet is an important driver—over genetics and other environmental factors—shaping the human gut microbiota (GM). The GM refers to the ecosystem of microorganisms (viruses, fungi, protozoa, archaea, and, in greater proportion, the bacteria) that reside in symbiosis, both in the small intestine and in the host colon [9]. Growing evidence in the scientific literature is employing GM baseline information in integrative models to follow dietary interventions since some types of foods serve as substrates for microbial growth, which modulates not only fecal composition but reflects host homeostasis and indicates the early emergence of metabolic disruptions, such as cardiovascular diseases [10] and liver steatosis and obesity [11], as well modulating the immune system [12].

Considering that fecal samples are easy to collect and being a non-invasive method, there is an important gap in the knowledge about the usefulness of the fecal microbiota to generate nutritional biomarkers. Although there are pioneer findings highlighting the role of the gut microbiome as a predictor of dietary response, there are few controlled studies that specifically evaluate the potential use of GM composition as a biomarker of food consumption. Furthermore, analysis of the GM has been focused on dysbiosis, which corresponds to adverse qualitative and/or quantitative changes in intestinal microorganisms, closely associated with pathological processes, or, less frequently, on eubiosis, related to the balance between beneficed and pathogenic populations affecting intestinal health [13], but not examining the role of fecal bacteria as a possible biomarker of food intake. Currently, for fecal samples, composition of GM can be found out using novel techniques, such as analysis of the length of the terminal restriction fragment directed to 16S rRNA (gene) [14] and nanopore; although pyrosequencing and next-generation sequencing are the preferred analytical methods, their analyses are challenging for routine clinical practice [15]. In this regard, the aim of this review was to summarize the available scientific evidence concerning gut bacteria associations with dietary intake and analyze the potential of GM composition as a sensitive marker of food/nutrient consumption and dietary adherence assessment.

## 2. Traditional Methods for Food Intake Recording

Evaluating and monitoring food intake in individuals or populations is habitually achieved by non-invasive practical methods involving diverse food registration tools from face-to-face consultation or through digital instruments [4]. Food intake computing and applicability cover both individual patient care as well as public health research, facilitating an understanding of the nutritional effects in health–disease mechanisms [16] and contributing to design nutritional strategies to combat diseases associated with unhealthy food intake and nutrition [17].

These methods usually require self-report, good recent memory, and available time for data recording [4]. In addition, the interpersonal understanding variations on the requested information, the motivation of the participants, and individual’s inherent culture and data misinterpretation result in a challenge by exposing the methods to measurement errors, reducing their reliability and reflection of reality [18]. Some traditional methods of assessing food consumption are food recall for the last 24 h, food frequency questionnaires, and food diary stand out [4].

The food recall of the last 24 h usually considers what the individual ingested the day before or in the 24 h of non-consecutive and random days, but it is not feasible for all populations because it is subjective and depends on recent memory. In addition, they generally require the assessment of food preparation, amounts ingested, and time between determinate meals, for example, which leads to a great intra- and interpersonal variability on dietary intake [19].

On the other hand, food frequency questionnaires evaluate habitual intake over a longer period (weeks, months) and deal with the frequency that a person consumes food items (1–3×/week, for example), classifying them into categories, associated with nutritional compounds. This tool can be qualitative, quantitative, or semi-quantitative, but, as disadvantages, relies on personal cooperation, is extensive, and does not assess the exact amount of nutrients ingested in a consistent manner [4].

In this context, the food diary comprises a gentle method, which depends on the participant’s motivation, covers the registration of all foods, beverages, and dietary supplements that an individual consumes within an established period, and can vary between days and months. Preferably, data should be recorded based on measurement in grams or milliliters of food portions, which leads to the need for prior training of the participants [17]. Together, the current methods of estimating food consumption, despite some benefits, such as low cost, practicality, and being non-invasive, have biases that compromise results’ value and suitability, emphasizing the need for complementary methods that accurately estimate nutrient intake, which can be detailed approaches using specific and validated metabolomic or metagenomic strategies.

## 3. Fecal Bacteria as a Biomarker of Health and Disease

**Figure 1:**
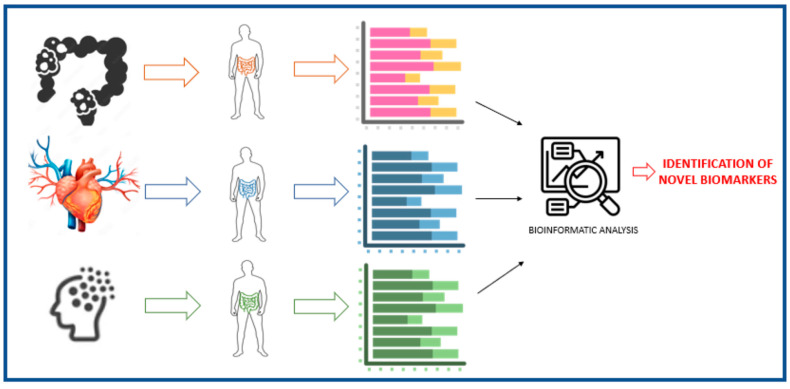
Gut microbiota as a biomarker of disease.

The human gut harbors communities of microorganisms, which play a crucial role in physiological and metabolic functions [9]. These microbes form a very complex ecological entity that interplay in many aspects with nutrition and health, such as transformation and production of metabolites, enzymes and vitamins, and extraction of nutrients from food [20]. The balance between beneficial versus pathogenic microorganisms, within intestinal and immunological homeostasis, is known as eubiosis. In contrast, qualitative and/or quantitative changes in microbial populations associated with loss of intestinal epithelium integrity and local and systemic inflammatory process are considered as dysbiosis [13]. Dysbiosis can alter the normal beneficial contribution by the microbiota to the host, as well as make the intestinal epithelium susceptible to pathogenic agents and molecules, leading to the fragility of the intestinal epithelial barrier, which is associated with systemic chronic inflammatory processes that favors the appearance of chronic non-communicable diseases (Figure 1) [20].

The microbiome is sensitive to many factors that can disturb balance (including infections, change in diet, and long-term use of antibiotics, stress, sleep disturbances, etc.), making an individual predisposed to disease, and can influence metabolic health through several interactions between the host and microbes [21], either mediated indirectly (through the availability of diet-dependent metabolites) or directly (through modulation of microbiome composition and post-biotic products) by diet [22]. However, the standard definition of a basal or healthy level for bacterial taxa, as well as general microbiota markers, is still evaluated based on abundance and richness (which are related to the total number of bacterial species and their characteristics in a sample), alpha diversity (related to the distribution of species abundances in a sample), and beta (which assesses the similarity between microbial communities) where Chao and Shannon indices are widely used for these purposes [23].

### 3.1. Gut Microbiota in Obesity

Overweight is a growing global health problem associated with several clinical comorbidities and impaired quality of life, whose etiology is multifactorial [24], being characterized by an excessive accumulation of white adipose tissue and accompanied by endocrine and inflammatory disturbances [25]. In recent years, GM has been associated with obesity installation, not only in adults but also in children [26]. Some investigations have reported the association of certain bacteria with obesity. In short, although some results show the highest proportion of Firmicutes in relation to Bacteroidetes in obese individuals, these findings are still controversial [27,28,29]. Similarly, there is a higher concentration of *Lactobacillus* spp. and a low proportion of *Bacteroides vulgatus*, in addition to an association between *Staphylococcus* spp. with the largest energy stock [30]. In contrast, *Akkermansia muciniphila* is reduced in the microbiota of obese individuals [31], stressing that some of the causal relationships or related consequences between obesity and fecal microbiota need to be verified.

### 3.2. Gut Microbiota in Weight Loss Response

The association between GM and host metabolic health is close, where changes in body weight have been shown to be accompanied by shifts in gut microorganism diversity in adults [32] and adolescents [33]. As an example, the genus *Akkermansia* has been widely associated with lean individuals and appears to be significantly more prevalent after weight reduction [34].

A study by Korpela et al. [35], applying regression models, successfully predicted host and microbiota responses to a weight control diet in obese patients, using the pretreatment abundance of fecal microbiota (mainly Firmicutes) as predictors. Another study showed that baseline GM was an important factor in determining diet-induced individual weight loss, where the abundance of *Blautia wexlerae* and *Bacteroides dorei* were the strongest predictors for weight loss [36]. Interestingly, a study by Christensen et al. [37] suggested that adults following the same diet, depending on baseline abundance levels of the *Prevotella* species in their gut, may lose more or less weight. In fact, these authors showed that adding more daily dietary fiber, without any caloric restriction, can lead to more weight lowering in individuals with a high abundance of *Prevotella*. In this line, similar results were obtained in other publications from the same group, where individuals with a high abundance of *Prevotella* lost more body fat after a new Nordic diet (rich in grains/fiber) than the standard Danish diet. Furthermore, fat loss was not observed in those with a low basal abundance of *Prevotella* species following the new Nordic diet [38].

Indeed, different nutritional strategies are used to promote weight and body fat reduction. However, the repercussions of the nutritional strategy can modify and benefit the host microbiota in different ways, depending on whether the person is male or female. This finding was observed in the study of Cuevas-Sierra et al. [39], which found, by offering a calorie-restricted diet, moderately rich in proteins for 4 months to overweight men and women, different responses concerning microbial abundance observed through metabolomic evaluations, with a significant decrease in class *Negativicutes* and species *Dielma fastidiosa* in men, while an increase was found in the species *Phascolarctobacterium succinatutens* and *Ruthenibacterium lactatiformans* in women.

These investigations show the role of GM as a biomarker of weight loss and suggest the evaluation of fecal composition and metabolites as potential predictors of metabolic responses and weight-lowering success, highlighting the need to establish models to individualize slimming diets prescription based on the composition of basal GM.

### 3.3. Gut Microbiota in T2DM

Excess weight and dysbiosis are closely associated with the chronic low-degree inflammatory process, which affects the production of inflammatory cytokines (IL-6 and TNF-α) and compromises the sensitivity and actions of hormones, such as insulin, contributing to insulin resistance and the onset of Type 2 Diabetes Mellitus (T2DM) in the longer term [40]. Among the findings reported after the analysis of the microbiota of subjects with T2DM, the genera *Bifidobacterium, Bacteroides*, *Faecalibacterium*, *Akkermansia*, and *Roseburia* are in smaller proportions, while the genera *Ruminococcus, Fusobacterium*, and *Blautia* are positively associated with the disease [41].

### 3.4. Gut Microbiota in Cardiovascular Disease

Microbial metabolism of the intestine relates to cardiometabolic homeostasis in different ways, where exacerbated production of metabolites, such as trimethylamine N-oxide or short-chain fatty acids, and changes in bile acid metabolism pathways seem to contribute negatively to cardiovascular health [42]. In this context, the abundant presence of *Porphyromonas gingivalis*, *Helicobacter pylori*, and *Chlamydia pneumoniae* is associated with atherosclerosis [43]. Likewise, this diseased population has an increased concentration of the genera *Collinsella*, *Roseburia*, and *Eubacterium* and butyrate-producing bacteria [44]. In addition, patients with atherosclerotic plaque have typical microbiome patterns with high levels of Proteobacteria and low levels of Firmicutes [42].

### 3.5. Gut Microbiota in Intestinal Diseases and Colorectal Cancer

The etiology of IBD (intestinal bowel disease) is partly attributed to a dysregulated immune response involving gut microbiome dysbiosis [45]. Multiple studies have documented differences in the composition of GM between patients with IBD and healthy individuals, particularly regarding microbial diversity and relative abundance of specific bacteria. Some of these bacteria are *Ruminococcus gnavus* (enriched), *Faecalibacterium prausnitzii*, and *Prevotella copri* (depleted) [46].

Additionally, the relative abundance of some taxa appears to correlate with established markers of this disease. In this sense, specific bacterial species, such as *F. prausnitzii* and *Clostridium difficile* (strongly accompanying dysbiosis, colitis, and severe diarrhea in humans), have been closely associated with IBD and proinflammatory responses, reinforcing their clinical value as a potential bacterial biomarker of this disease, as assessed by the presence of *F. prausnitzii* and *Escherichia *coli** in 28 healthy controls, 45 patients with CD, 28 patients with UC, and 10 patients with IBS [47]. Additional findings from these patients confirmed that *F. prausnitzii* was a specific indicator of IBD and was significantly lower [48].

Some further evidence indicates that GM plays a vital role in the initiation, progression, and metastasis of colorectal cancer [49]. In the same way, the scientific literature has been expanding the knowledge about bacterial populations that, when in excess, are associated with their development, highlighting the presence of *Streptococcus bovis, Bacteroides fragilis enterotoxigénicos*, *Fusobacterium nucleatum*, *Enterococcus faecalis*, *E. coli*, and *Peptostreptococcus anaerobius* as main pathogens [50].

### 3.6. Gut Microbiota in Mental Diseases

In the last few years, growing evidence has pointed towards the bidirectional gut microbiota–brain axis playing a role in mental health [51,52,53]. The current scientific data support an altered gut microbiome in subjects with mental disorders, such as depression and anxiety, and point to some bacterial components as potential biomarkers related with these diseases. Thus, in the Flemish Gut Flora Project, fecal *Dialister* and *Coprococcus* spp. were markers of good mental health [54]. On the other hand, Heym et al. studied 40 participants from the general population in the UK and found that the fecal abundance of *Lactobacillus* spp. was directly related to positive self-judgment but only indirectly to cognitive depression and lower affective empathy [55].

Other studies have revealed the role of genera, such as *Coprococcus*, *Bifidobacterium*, *Lactobacillus*, *Roseburia*, and *Faecalibacterium*, with lower levels of anxiety and depression [56]. In fact, *Bacteroides*, *Escherichia*, *Shigella*, and *Streptococcus* are associated with higher levels of stress [57]. In addition, the genus *Eggerthella* (and, in general, the depletion of certain anti-inflammatory butyrate-producing bacteria) appeared to be shared between major depressive patients [58]. The study of Lucidi et al. [59] showed the potential role of *Pseudomonas aeruginosa* as a possible biomarker for discriminating patients with affective disorders from control individuals. Further, these authors found that the Lachnospiraceae family might play a role in the onset of depression via affecting the inflammation levels in the host.

## 4. Gut Microbiota and Food Intake

**Figure 2:**
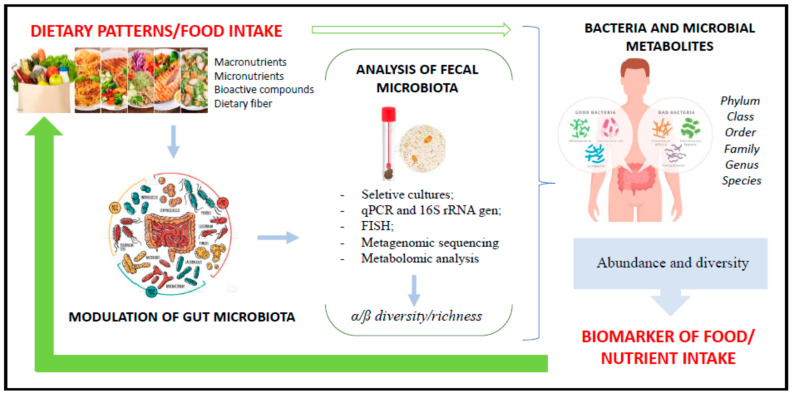
Use of bacterial microbiota as a biomarker of food intake.

Dietary patterns are recognized to be involved in disease and health [50,51,52,53,54,55,56,57,58,59,60,61,62]. However, the impact of different foods and dietary patterns on the modulation of the microbiota is not yet clearly elucidated but is known to drive changes in GM [62], intestinal barrier functions, and immune system competence [63,64]. The scientific literature shows that not only sex, age, physical activity, and other lifestyle factors influence GM but that 3 days of dietary interventions (composition and mealtime) are already able to induce changes in bacterial composition and even alter the set of postbiotic molecules that microbes produce [65,66,67]. Thus, the diet is recognized as a key modifiable factor in the manipulation of the microbial community, with a direct impact on the composition and maintenance of beneficial bacterial populations through the continuous supply of dietary substrates [62]. In this context, recent research found a microbiota pattern or signature associated with different dietary patterns, and these results drive a new possibility to use GM not only as associated to diseases [68,69] but also as a biomarker of dietary intake (Figure 2).

Recently, PREDICT 1 (Personalized Responses to Dietary Composition Trial 1) [70] was able to study the gut microbiome on a scale and complexity never seen before. Through metagenomic sequencing (average of 8.8 ± 2.2 gigabases/sample), along with long-term dietary data and hundreds of measurements of participants’ fasting and postprandial blood markers, it was possible to identify a set of microbial species that are strongly and consistently linked to cardiometabolic biomarkers and related to obesity and postprandial responses, as well as to dietary patterns, approximating the analysis of the GM for precision clinical practice [70] and consistent use as food intake as a biomarker. Indeed, dietary patterns may display characteristic microbiome signatures depending on the composition and nutrient distribution (Table 1).

### 4.1. Gut Microbiota and Dietary Patterns

#### 4.1.1. Mediterranean Diet

The Mediterranean diet (MD) is characterized by daily consumption of whole grains/pulses and cereals (fiber and carbohydrates), legumes, vegetables, and fruits; mono- and polyunsaturated fatty acids (extra virgin olive oil and oilseeds); bioactive and antioxidant compounds, as flavonoids, phytosterols, terpenes, and polyphenols [85], in addition to discouraging the consumption of excessive red meat and saturated fat and moderating the consumption of dairy products [71], whose nutritional composition pattern partly mimics the Dietary Approaches to Stopping Hypertension (DASH diet), which produces positive effects in the prevention and control of cardiovascular and other metabolic diseases [72,86,87].

MD positively modulates the host microbiota, leading to different local and systemic responses, correlating with the re-establishment of eubiosis [88] concerning the Bacteroidetes and beneficial groups of *Clostridium*, with a detriment on the Proteobacteria phylum and Bacillaceae family levels [89]. In 2018, Garcia-Mantrana [71] observed, in adults with a high adherence to MD, that GM was composed of 77.31% ± 2.88 of Firmicutes, 15.86% ± 0.28 of Bacteroidetes, 3.13% ± 0.65 of Actinobacterias, 1.78% ± 1.22 of Verrucomicrobia, and slightly less than 1% of Proteobacterias. In investigations conducted through the PREDIMED program (Prevención con Dieta Mediterránea) [87], it was found that adherence to MD had a lower consumption of animal-protein-associated higher concentration of Bacteroidetes. At the same time, participants who consumed more complex carbohydrates and plant proteins produced higher amounts of volatile short-chain fatty acids [87].

The intake of oleic acid derived from extra virgin olive oil when consumed in excess may have an unfavorable effect on the bacterial diversity of GM [73]. However, MD daily consumption, in adequate amounts for each individual, is associated with an increase in lactic-acid-producing bacteria, mainly *Bifidobacterium* and *Lactobacillus*, leading to reductions in inflammatory cytokine secretion (IL-6, IL-17A, TNF-α, IL-1β, COX-2, LDC-LDC) [90] and the stimulation of butyrate production, with anti-inflammatory and atheroprotective actions, defending colonocytes against oxidative stress [91].

The GM is favored by the consumption of another typical MD component, such as omega-3 fatty acids, which has a repercussion in the balance of the proportion of Firmicutes:Bacteroidetes and increased bacteria of the family Lachnospiraceae and genus *Bifidobacterium*, while controlling the presence of lipopolysaccharides and Enterobacteriaceae family, with potential anti-inflammatory effects [92]. In another way, the high availability of polyunsaturated fatty acids acquired by the diet seems to inhibit some bacterial populations, reducing the risk of obesity and inflammation [74].

The *Roseburia* spp. is an important member of the microbiota that metabolizes omega-6 fatty acid and converts it into conjugated linoleic acid, which is recognized by immune cells, enhancing the function of regulatory T cells [93]. Likewise, *Lactiplantibacillus plantarum* is known for producing conjugated linolenic acid, eliciting an important impact on the composition of the microbiota by stimulating the trophic presence of *Ruminococcus* and *Prevotella*, leading to a reduced level of pro-inflammatory cytokines and increased IL-10 (anti-inflammatory) and nuclear peroxisome proliferator-activated receptor-γ (PPAR-γ) [93].

#### 4.1.2. Plant-Based Diet

Plant-based diets include vegetarian and vegan patterns involving a low consumption of animal proteins (from fish, eggs, and dairy products) or no animal food consumption, respectively [83]. The abundant supply of fruits, vegetables, whole grains, pulses, seeds, oils, and vegetable fats constitutes an important source of dietary fiber and bioactive compounds [94]. The composition of GM among vegans and vegetarians may not differ, and both include a higher composition of beneficial fecal bacteria when compared to omnivores [95]. Thus, research data show that plant-based diets are associated with high fecal levels of species of genus *Prevotella* [41,96], which has anti-inflammatory properties [87]. In a study by Filippo et al. [97], it was possible to verify that the GM of children from Burkina Faso (Africa), who had a diet based on vegetables (rich in fiber and resistant starch), when compared to children from Italy, who had a diet like the Western (low in fiber), elicited relevant differences in bacterial phylum count: Actinobacteria and Bacteroidetes were more represented in Africa than in Italian children (10.1% versus 6.7% and 57.7% versus 22.4%, respectively), whereas Firmicutes and Proteobacteria were more abundant in Italian than in African children (63.7% versus 27.3% and 6.7% versus 0.8%, respectively).

Moreover, in an experimental study with rodents, some effects of the plant-based diet on GM were tested, where there was a significant increase in genus *Bacteroides* and *Alloprevotella*, and a reduction in genus *Porphyromonas* and *Erysipelothrix* [76]. Similarly, diets rich in complex carbohydrates, whole grains and wheat bran were associated with increased *Bifidobacterium* spp. and *Lactobacillus* spp., which play a protective role in the intestinal barrier by inhibiting the invasion and growth of pathogens [98]. Likewise, resistant starch and whole barley can also increase lactic acid bacteria (*Ruminococcus* spp., *Eubacterium rectale*, *Roseburia* spp.), apparently benefiting the systemic health of the host [77].

Thus, plant-based diets and associated main food components affect the bacterial composition and metabolic pathways of the GM positively, increasing symbiotic microorganisms and favoring global health [99]. However, more studies are needed to determine the impact of these diets on intestinal microbes since, nowadays, the use of chemicals to favor the growth, maturation, and conservation of food can compromise putative benefits on GM.

#### 4.1.3. Western Diet

Western diet (WD) consumption represents a global health concern because it is related to increasing rates of obesity and chronic non-communicable diseases, characterized by high caloric density associated with frequent consumption of unhealthy fats (saturated and trans), refined sugars, salt, alcohol, and other elements, such as dyes, preservatives, and antimicrobials, and also with reduced consumption of fruits, vegetables, and legumes, among other foods [78].

The adoption of this dietary pattern seems to have distinct repercussions on the microbiota of men and women [42], although it is already associated with dysbiosis, enterocytic dysfunctions, and increased intestinal permeability, in addition to the leakage of toxic bacterial metabolites into the circulation, contributing to the development of low-grade systemic inflammation [79]. When evaluating GM in consumers of WD, there is a reduced overall count of microorganisms and a change in the abundance of bacterial species. In general, the study of the Firmicutes to Bacteroidetes ratio has been linked to Western diet consumption and obesity, which seems to be accompanied by an increased abundance of class Erysipelotrichales and Bacilli [67].

In a meta-analysis performed by Jiao et al. [100], it was found that the relative abundance of Actinobacteria is reduced and that there is an increase in Proteobacteria. Additionally, the dominance of four bacterial classes (Bacteroidia, Clostridia, Bacilli, and Erysipelotrichi) was observed, corresponding to 90% GM composition for a high-fat diet (HFD). Likewise, a HFD is associated with reductions in some fecal populations, such as Prevotellaceae, Rikenellaceae, and *Bifidobacterium* spp., which is negatively correlated with the function of the intestinal barrier [101]. Interestingly, when assessing the fecal sample of men and women in the Spanish population, with a high frequency of consumption of ultra-processed foods (>5 servings/day), it was possible to demonstrate associations between increases in *Bifidobacterium* and Actinobacteria with the consumption of pizza and Actinobacteria with industrialized dairy in women. For men, it was reported that an increase in Bacteroidetes correlated positively with processed meat [102]. Despite the findings that support the negative impact of the WD on GM, the cause for which these changes occur is still inconclusive, since studies are conducted with different types, amounts, and proportions of fats, sugars, calories, and dietary fiber, impacting microbial health.

### 4.2. Gut Microbiota and Nutrient Intake

#### 4.2.1. Carbohydrate and Dietary Fiber

Carbohydrates are a main group of macronutrients which yield energy, being chemically categorized into non-fibrous polysaccharides, lignin, resistant starch, and non-digestible oligosaccharides/dietary fibers (DFs) [103]. DFs are assigned according to insoluble and soluble properties and are often abundant nutrients in both plant-based and omnivorous diets, by consuming foods, such as cereals, roots and tubers, legumes, fruits, and vegetables [104].

Soluble fibers elicit a prebiotic effect, being rapidly metabolized and fermented by intestinal bacteria, significantly influencing the abundance and diversity of GM [105]. At the same time, the undigestible oligosaccharides are resistant to digestion in the small intestine and pass to the colon, where they are exposed to bacterial utilization being affected by the type, number, and colonization of intestinal bacteria, with beneficial effects already reported on *Bifidobacterium* and *Lactobacillus* levels, favoring the production of short-chain fatty acids (acetate, butyrate, and propionate) [106] and inhibiting the growth of some intestinal pathogens of the Enterobacteriaceae family (*Salmonella* spp., adherent-invasive *Escherichia coli*), as reported [76].

Moreover, modern dietary patterns are associated with a high intake of refined carbohydrates, such as fructose, mainly found in the form of corn syrup in beverages and ultra- and processed foods, with a reduced consumption of dietary fiber. Together, these changes negatively impact bacterial diversity and survival, leading to dysbiosis [80] and non-alcoholic fatty liver disease [11]. In insufficient fiber consumption, intestinal bacteria resort to glycoproteins of the mucus layer. However, only a few species can use this source of nutrient (such as the species *Bacteroides thetaiotaomicron*), which reduces bacterial diversity and associated potential benefits [81,107]. Additionally, there are some practices based on nutritional strategies, such as a low-carbohydrate diet (low-carb diet), ketogenic, and low FODMAPS (fermentable oligo-, di-, monosaccharides, and polyols), which are designed to reduce dietary sources of carbohydrate and dietary fiber [82,83,84]. In general, low-carb diet adherence leads to a reduction in the abundance and diversity of beneficial bacteria, with a fall in Firmicutes (mean abundance: 5.53), Verrucomicrobia (mean abundance: 0.51), *Eubacterium rectale*, *Dialister*, *Ruminococcus gnavus*, and *Clostridium* accompanying an increase in *E. coli*, *Desulfovibrio* spp., *Parabacteroides*, and Bacteroidetes (mean abundance: 5.29) [63].

#### 4.2.2. Fat

Dietary fats are macronutrients that, in addition to providing energy, are essential for some metabolic pathways, such as the transport of fat-soluble vitamins, cell membrane composition, and hormonal synthesis [108]. Lipids can be found in the form of unsaturated fat (mainly mono- and polyunsaturated), saturated, and produced by the food industry in the form of trans fatty acids [109].

The high intake of saturated fats and omega-6 polyunsaturated fatty acids or small amounts of omega-3 and an omega-6/omega-3 ratio of 20:1 has been related not only with adverse metabolic consequences but also with changes in the GM [91]. Dysbiosis linked to excess dietary fats is commonly associated with weight gain and has repercussions, such as reduced total count of intestinal microorganisms, change in the abundance of bacterial species, and progression of intestinal permeability [110]. Changes in GM depend on the type of fatty acids ingested, where the intake of omega-3 is directly associated with an increase in the abundance of *Lactobacillus*, while monounsaturated fatty acid and omega-6 consumptions are inversely related to *Bifidobacterium* content [111].

In addition, changes in microbiota composition induced by a high-fat diet in animal and human models mainly favor an increase in the proportion of Firmicutes to Bacteroidetes (73% and 21%, respectively) [79]. On the other hand, another study noted an increase in dietary fat in the short term produced increases in *Alistipes* and *Bacteroides* [67]. Likewise, a rise in the abundance of Proteobacteria phylum and a fall in the levels of *Prevotellaceae* and *Rikenellaceae* family were also found, as well as a reduction in *Bifidobacterium* spp. after high fat intake [100].

Indeed, the amount of fat in the diet is an important driver of microbial fecal oscillation, with direct relationships with the metabolic homeostasis of the host, thanks to the unregulated modulation that fat exerts on the Reg3γ (regenerating islet-derived protein III gamma), which consequently and negatively influences the abundance and endogenous variation in bacterial species, leading to dysbiosis [112]. Intriguingly, some results are inconsistent in relating different proportions and types of fat sources with changes in the microbiota, which seems to be justified by the different amounts of dietary fiber offered in the diets, a putative conflicting factor in the evaluation of the cause–effect relationship between dietary fat and GM [113].

#### 4.2.3. Protein

Proteins are a macronutrient that supply important substrates, such as amino acids, and often play a precursor role in the synthesis of enzymes, antibodies, and muscle deposit. Animal or vegetable protein sources vary according to the composition of the peptide chain and supply of amino acids (essential and non-essential). In this context, GM plays an essential role in amino acid metabolism, both in the small intestine and in the gut [114], where proteins are hydrolyzed by proteases and peptidases secreted by gut bacteria in the intestinal lumen, which may be absorbed by enterocytes or fermented by bacterial species in short-chain fatty acids, hydrogen sulfate, and ammonia [115].

Vegetable proteins often have low digestibility [116], while animal protein is more easily degraded by aerobic microorganisms in the large intestine, with a lower incidence of gastrointestinal effects [117]. In the small intestine, bacterial populations, such as *Klebsiella* spp., *Escherichia coli*, *Streptococcus* spp. *Succinivibrio dextrinosolvens*, *Mitsuokella* spp., and *Anaerovibrio lipolytica*, directly metabolize amino acids and can secrete various proteases and peptidases [116,117]. Protein molecules and undigested peptides are fermented, resulting in the production of microbial metabolites, such as short-chain fatty acids, ammonia, polyamines, hydrogen sulfide, phenolic, and indolic compounds, which can be transported to colonocytes and elicit beneficial or deleterious effects on epithelial cells, depending on their concentrations in the lumen [118].

In the colon, bacterial genera *Bacteroides* and *Clostridium*, and phylum Proteobacteria, which are potentially pathogenic, are related to protein substrates from animal sources, particularly from red meat and dairy products [79], and produce toxic substrates, such as ammonia and polyamines, which include nitrosamines and trimethylamine N-oxide [119], implicated in cardiovascular disorders [120]. Thus, when the consumption of this type of protein becomes excessive, it is necessary to reduce these potential pathogens and consequently restore the microbial ecosystem through the change in dietary composition [121]. In contrast, plant proteins, especially from soybeans and peanuts, may play a positive role in modulating beneficial bacterial composition in the intestine, increasing communities of *Bifidobacterium* and reducing Enterobacteriaceae family and *Clostridium perfringens* in rats after nitrogenous enrichment of the diet with 20% peanut protein [122].

#### 4.2.4. Micronutrients: Vitamins and Minerals

Studies relating GM to a single micronutrient are rare, since the food itself is composed of a set of nutrients [123]. However, experimental studies using the daily supplementation of isolated micronutrients demonstrated a crucial role in the regulation of energy metabolism, growth, cell differentiation, and immune functions, including possible methods of interaction with fecal microbiota composition [124,125].

Interestingly, some vitamins are synthesized by GM (thiamine, riboflavin, niacin, biotin, pantothenic acid, folate, or vitamin K) through the mediation of various intestinal bacteria, such as the phyla Bacteroidetes, Fusobacteria, and Proteobacteria [126]. On another side, sun exposure and vitamin D supplementation were associated with increased *Lachnobacterium* and reduced *Lactococcus* in children aged 3 to 6 months [127]. Vitamin K can be acquired through dietary sources and through bacterial fermentation, with the consequent production of menaquinone [128]. Recently, it was observed, in a study with rodents, that low intake of this vitamin is associated with changes in the microbial composition of the intestine and that dietary supplementation of vitamin K leads to an increase in the family Lachnospiraceae FCS020 and Ruminococcaceae UCG-009 in females and increase in the genus *Ruminococcus_1* in males, favoring bacterial diversity [129].

Furthermore, upon reaching the colon, some vitamins positively modulate GM. In 2019, Choi et al. [130] analyzed the impact of different dosages of vitamin E on the composition of GM and found that its deficiency is related to a proportion of 61% of Firmicutes, 36% of Bacteroidetes, 0,5% of Verrucomicrobia, and 1.3% of Proteobacterias. Vitamins A, B2, D, and beta-carotene lead to increased abundance of bacterial species; vitamins A, B2, B3, C, and K maintain microbial diversity; vitamin D favors the richness and diversity of microorganisms and vitamin C leads to increased production of short-chain fatty acids. Additionally, the impact of vitamins A and D is also reported on the modulation of the intestinal immune response, with secondary repercussions on gastrointestinal health and microbiome [131,132].

Regarding minerals, it has been evidenced that iron is a key element involved as a cofactor in redox reactions, diverse metabolic pathways, and electron transport chain mechanisms but also to influence the composition of the microbiota [132]. Thus, Constante et al. [133] demonstrated, in mice, that a diet rich in heme iron favored the abundance of Proteobacteria and reduced the abundance of Firmicutes. Another trial with rodents reported that excessive sodium intake is associated with reduced abundance of *Lactobacillus* spp. and the genera *Oscillibacter*, *Pseudoflavonifractor*, *Clostridium* clusters XIVa, *Johnsonella*, and *Rothia*, while greater abundance of *Parasutterella* spp. and *Erwinia* species, and the families Christensenellaceae, Corynebacteriaceae [134], Lachnospiraceae, and Ruminococcus [79]. In particular, a reduction in *Lactobacillus* spp. associated with excess sodium consumption increased Th17 cells and favored the expression of pro-inflammatory processes by altering intestinal homeostasis and reflecting increased vulnerability to inflammatory insults [135].

#### 4.2.5. Bioactive Compounds and Probiotics

Bioactive compounds (BCs) are characterized as chemical molecules acquired through dietary or external supplements where, although not essential for survival or produced by the human body, their intake confers benefits [136]. These compounds, with wide structural diversity, are widely found in food sources in the plant kingdom [137]. BCs consist of flavonoids, phenolic acids, stilbenes, lignans, and many others and, when ingested, a low proportion is absorbed in the small intestine, while habitually, the largest amount remains in the colon and is metabolized by gut bacteria [137].

The interaction between the consumption of BCs and GM is bidirectional: in one strand, it was found that bacterial fermentation is an essential process that directly influences the bioavailability and bioactivity of the BCs and, on the other hand, BCs may modulate the composition of GM thanks to the action of their aromatic or other metabolites [138]. Dietary polyphenols are widely studied bioactive components that increase both *Bifidobacterium* spp. and *Lactobacillus* spp., providing cardiovascular protection, with antibacterial and anti-inflammatory effects [75]. Similarly, in the study of Molan, Liu, and Plimmer [139], humans that received carotenoids through the ingestion of blackcurrant (672 mg/day for 2 weeks) induced an increase in *Bifidobacterium* spp. and *Lactobacillus* spp. and a reduction in *Bacteroides* spp. and *Clostridium* spp.

Further, in an experimental trial, rats receiving a high-fat diet and synthetic fructose were supplemented with pterostilbene (15 or 30 mg/kg), which showed increased abundance of *Akkermansia* and *Erysipelatoclostridium* at the same time as a decrease in *Clostridium* [140]. In studies with animals, there is a divergence of results due to methodological variability. Thus, the consumption of anthocyanin seems to reduce the phylum Verrucomicrobia [141] while the consumption of polyphenols increases the concentrations of *Akkermansia muciniphila* [142]. Furthermore, flavonoid consumption was associated with a reduction in Firmicutes [143], while saponin intake increased this microorganism in fecal samples [144].

Kefir is a fermented product produced by a culture of lactic acid bacteria (such as *Lactobacillus harbinensis*, *Lactobacillus paracasei*, and *Lactiplantibacillus plantarum*), acetic, and yeasts that exert probiotic activity [145], with an influence on tolerance to bile acids and salts on adhesion of the intestinal mucosa and antimicrobial resistance, providing health benefits [146]. However, when evaluating its impact on the composition of GM, there is only an increase in the relative abundance of Lachnospiraceae *A2* (Linear Discriminant Analysis = 4.60) and reduced the relative abundance of the genus *Clostridium* and family *Clostridiaceae* (Linear Discriminant Analysis = 4.25), which suggests the need for further studies [147]. In summary, the intake of BC impacts GM diversity, with intestinal and systemic repercussions [10,70,96,148]. Currently, there is industrial manipulation of a multitude of probiotic strains, which colonize, survive, and differentiate in the gut environment according to the food stimuli. Thus, the intake of probiotic strains and their trophic action are directly related to the type of nutrient and diet ingested [149]. However, depending on the component, the dose consumed, and the method of preparation, the inhered repercussions on GM can be questionable and need further elucidation [150].

## 5. Fecal Microbiota as a Marker of Food Intake: Current Situation and Future Challenges

Interest in devising biomarkers of food and nutrient intake has been advancing rapidly in recent years, which has been driven by practical needs in proposing new methods for assessing and monitoring food intake. Understanding relationships will allow for the detection of dietary changes from their initial moment, which facilitates an early nutritional intervention, contributing to the prevention of chronic non-communicable diseases associated with food imbalances as well as the evaluation of dietary adherence during clinical treatments.

Metagenomic studies are playing an important role in the identification of biomarkers of food intake and represent a precise approach that reflects the physiological function driven by food intake. Healthy eating is associated with body homeostasis in all systems, which is based on the complex interaction between biochemical and physiological pathways at different cellular levels that are responsible for maintaining health, including GM.

Currently, robust nutritional intake biomarkers are scarce, impacting the delay concerning advances around nutritional and dietary assessment. However, it is already known that GM is directly modulated by the composition of the diet and that the isolated consumption of certain nutrients or food groups stimulates the growth of specific bacterial taxa, which, interestingly, suggests that the composition of intestinal bacteria is a potential mirror of food consumption.

The gastrointestinal tract is extensive and has distinct bacterial populations throughout anatomize portions, where the collection of fecal samples is an eventually practical, fast, and non-invasive method for the evaluation of the composition of bacterial species and their metabolites. Therefore, GM seems to be a viable tool for dietary assessment (Table 2).

Dietary patterns have an impact on GM (Table 1) and, among the different patterns, it is observed that those consisting of high dietary fiber and bioactive components intake are controlled in animal and dairy protein and reduced in ultra-processed consumption, such as the Mediterranean and vegetable diets, associated with greater abundance and diversity of bacterial groups, positively affecting lipid metabolism, inflammatory state, liver, intestinal function, and immune control through different metabolic pathways and epigenetic interactions.

On the other hand, the scarcity of dietary fiber, micronutrient deficiency, and the exacerbated consumption of refined sugars, saturated fats, and sodium negatively modulate this ecosystem, reducing bacterial diversity and loss of epithelial integrity in the intestine, which is associated with dysregulation of inflammation, body adiposity, increased expression of inflammatory cytokines, and the emergence of chronic non-communicable diseases, such as obesity and metabolic syndrome [12]. In addition, species, such as *Bifidobacterium* spp., *Lactobacillus* spp., and *Akkermancia muciniphila*, are already associated with host health [21,28,31] and, conversely, Bacteroidetes and *Ruminococcus* spp. show the unfavorable conditions at the core of metabolism and inflammatory state [42,61], emphasizing the direct relationship between diet and the composition of GM.

The heterogeneity between individuals/groups (sex, age, genetics, lifestyle, and others) and dietary variations among different populations, in addition to access to appropriate methodologies, constitutes a practical limitation in this area of study. However, as future perspectives, considering the number of data and valuable information that can be extracted from both GM and diet, the technological advancement, and the understanding of the cause/consequence relationships between gut bacterial species and diet should be considered.

**Figure 3:**
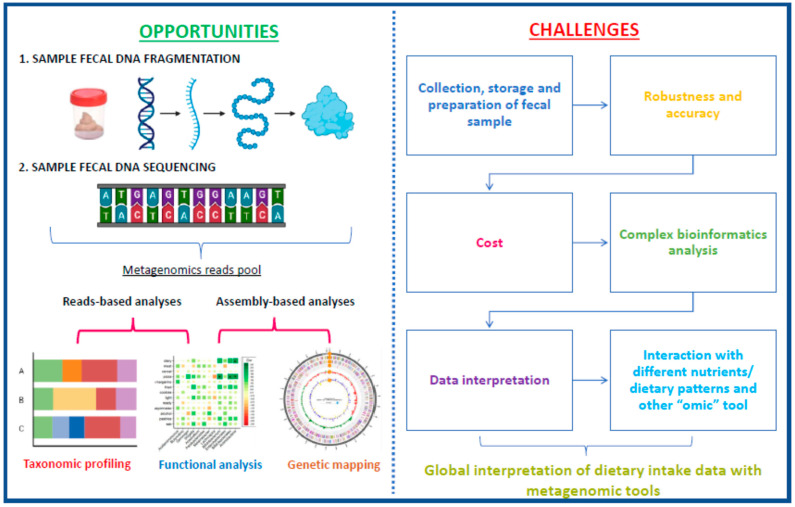
Opportunities and challenges for metagenomic-based biomarker development in fecal microbiota. Based on Milani et al. [151].

In this context, the relationship between food consumption and health status/ disease brings with it a new aspect of evaluation, where GM plays a central role. Thus, the study of bacterial composition (abundance/diversity), derived metabolites, and dynamics and their association with food intake emerges as a promising prediction tool of the “omics era” (Figure 3). This new view can facilitate the understanding of the repercussions of eating different dietary patterns and nutrients on metabolic health and inflammatory status and allows for the development of personalized and accurate nutritional strategies through GM modulation, with injected on personalized precision nutrition.

## Figures and Tables

**Table 1:** Characteristics of gut microbiota according to modern dietary patterns.

Dietary Pattern	Gut Microbiota	Referencing
Mediterranean diet	↑ Bacteroidetes, *Clostridium*, *Bifidobacterium*, *Lactobacillus* ↑ SCFA and diversity of MO ↓ Proteobacterias, Bacillaceae	[71,72,73]
Plant-based diet	↑ Bacteroidetes, *Prevotella* spp., *Xylanibacter*, *Bifidobacterium* spp., *Lactobacillus* spp., *Ruminococcus* spp., *Eubacterium rectale*, *Roseburia* spp. ↑ SCFA ↓ Firmicutes, Porphyromonadaceae, Erysipelotrichaceae	[38,74,75,76,77]
Western diet	↑ Firmicutes (Bacilli, Clostridiales), Erysipelotrichaceae, Proteobacterias, *Bacteroides thetaiotaomicron* ↑ LPS ↓ Actinobacterias, Prevotellaceae, Rikenellaceae, *Bifidobacterium* spp., Tenericutes ↓ Total count and abundance of bacterial species	[67,78,79,80,81]
Low-carb diet	↑ *E. coli*, *Desulfovibrio* spp., *Parabacteroides*, Bacteroidetes *↓* Firmicutes, *Akkermancia*, *Eubacterium rectale*, *Dialister*, *Ruminococcus gnavus*, *Clostridium*. ↓ Total count and abundance of bacterial species	[65,82,83,84]

LPS: Lipopolysaccharides. MO: Microorganisms. spp.: Unidentified species. SCFA: short-chain fatty acids. Low-carb diet: Low-carbohydrate diet.

**Table 2:** Selected gut microbiota according to dietary pattern/nutrient intake.

Selected of Gut Microbiota	Diettary Patterns/Nutrient Intake		Referencing
	Increase	Decrease	
Bacteroidetes	Mediterranean diet Plant-based diet Low-Carb diet Soluble fiber Vitamin A, B complex, C D, E, K Excess of animal protein	Western diet Excess of saturated fat	[63,71,76,89,103]
*Bifidobacterium* spp.	Mediterranean diet Plant-based diet Insoluble fiber Omega-3 Vegetable protein Polyphenols Vitamin A, B complex, C D, E	Western diet Omega-6 Excess of saturated fat	[90,97,100,104,105,107,111,122]
*Lactobacillus* spp.	Mediterranean diet Plant-based diet Insoluble fiber Omega-3 Omega-6 Polyphenols	Excess of sodium Excess of saturated fat	[75,94,139,145]
*Prevotella* spp.	Plant-based diet Omega-6 Vitamin A, B complex, C D, E	Western diet Excess of saturated fat	[41,94,97,100,102]
*Ruminococcus* spp.	Plant-based diet Omega-6 Vitamin K Excess of sodium	Low carb diet Excess of saturated fat	[63,77,79,94,129]
*Akkermansia* *muciniphila*	Mediterranean diet Plant-based diet Polyphenols Vitamin A, B complex, C D, E	Low carb diet Excess of saturated fat	[140,142]
*Clostridium* spp.	Mediterranean diet Excess of animal protein	Vegetable protein Excess of sodium Polyphenols	[62,89,135,139,140]
*Firmicutes* spp.	Western diet	Plant-based diet Low carb diet Polyphenols Heme iron	[62,67,76,92,133,143]
*Roseburia* spp.	Plant-based diet Omega-6 Vitamin K	-	[77,93,129]
Proteobacteria	Western diet Excess of animal protein Excess of sodium Heme iron	Mediterranean diet Plant-based diet	[78,79,133,134]
Enterobacteria	Western diet Excess of animal protein Excess of saturated fat	Mediterranean diet Plant-based diet Insoluble fiber Omega-3	[92,97,120]

spp.: Unidentified species. Low-carb diet: Low-carbohydrate diet.

## Data Availability

Not applicable.
